# Circuity analyses of HSR network and high-speed train paths in China

**DOI:** 10.1371/journal.pone.0176005

**Published:** 2017-09-25

**Authors:** Xinlei Hu, Shuo Zhao, Feng Shi, Jie Huang, Xinghua Shan

**Affiliations:** 1 School of Traffic and Transportation Engineering, Central South University, Changsha, Hunan, China; 2 Key Laboratory of Regional Sustainable Development Modeling, Institute of Geographic Sciences and Natural Resources Research, Chinese Academy of Sciences, Beijing, China; 3 Institute of Computing Technologies, China Academy of Railway Sciences, Beijing, China; Beihang University, CHINA

## Abstract

Circuity, defined as the ratio of the shortest network distance to the Euclidean distance between one origin–destination (O-D) pair, can be adopted as a helpful evaluation method of indirect degrees of train paths. In this paper, the maximum circuity of the paths of operated trains is set to be the threshold value of the circuity of high-speed train paths. For the shortest paths of any node pairs, if their circuity is not higher than the threshold value, the paths can be regarded as the reasonable paths. With the consideration of a certain relative or absolute error, we cluster the reasonable paths on the basis of their inclusion relationship and the center path of each class represents a passenger transit corridor. We take the high-speed rail (HSR) network in China at the end of 2014 as an example, and obtain 51 passenger transit corridors, which are alternative sets of train paths. Furthermore, we analyze the circuity distribution of paths of all node pairs in the network. We find that the high circuity of train paths can be decreased with the construction of a high-speed railway line, which indicates that the structure of the HSR network in China tends to be more complete and the HSR network can make the Chinese railway network more efficient.

## Introduction

In recent years, the rapid development of a high-speed rail (HSR) network in China has evolved into a large-scale HSR network. Each high-speed railway line is a reasonable train path. A HSR network consists of a number of railway lines. Among these lines, a passenger transit corridor can be found when the line not only meets a large passenger demand, but also has a low indirect degree of train path. On the high-speed railway network, except for trains running along each railway line, a large number of cross-line trains are operated. The operation of cross-line trains can decrease the transfer ratio and transfer times, and promote the service level drastically.

**Fig 1:**
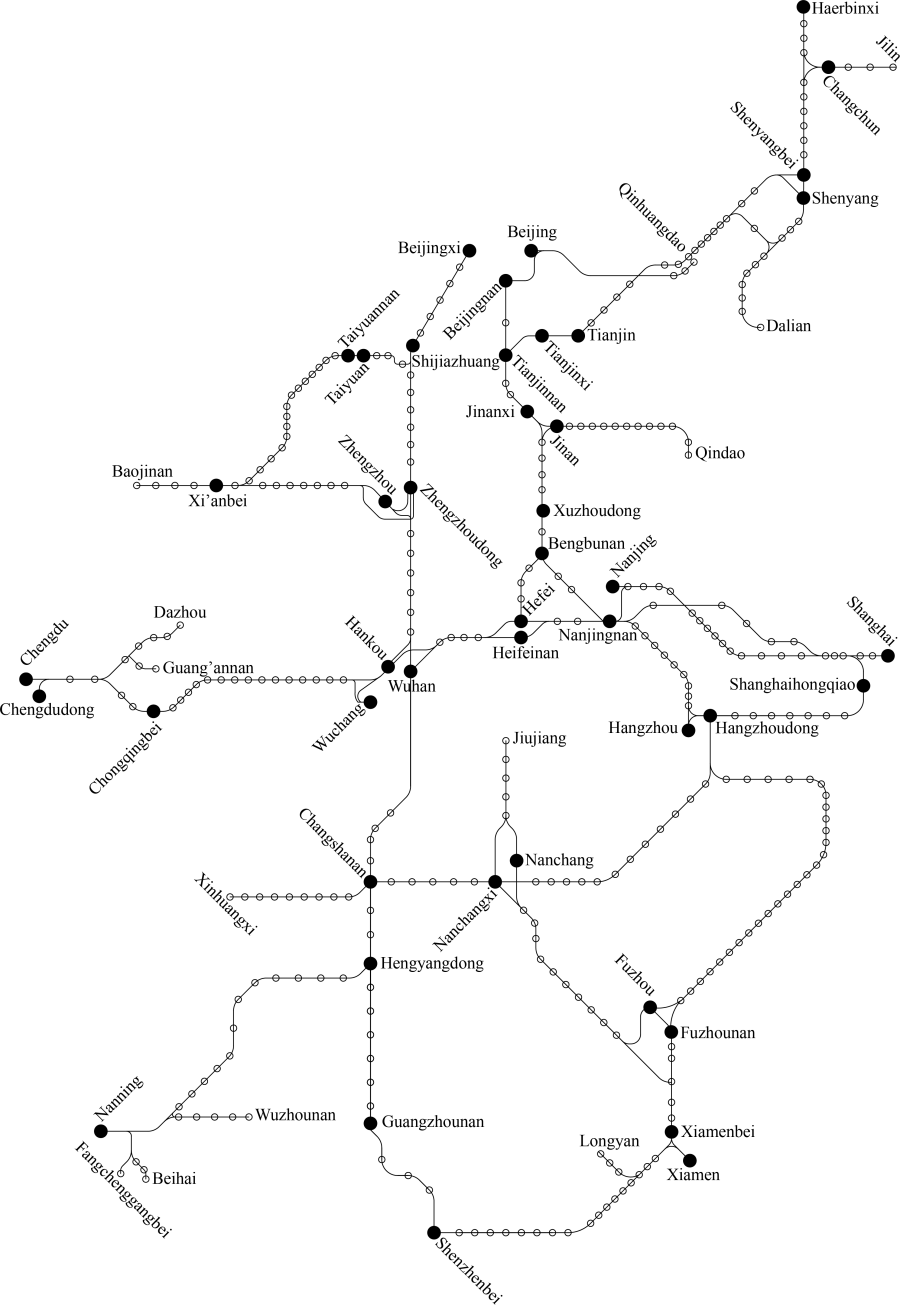
HSR network in China completed at the end of 2014 (reprinted courtesy China Railways Corp).

The construction of a railway line is constrained not only by geographical conditions along the line, but also by passenger demand. Although many conventional railway lines have been constructed along passenger transit corridors, high-speed railway lines may not be designed along all these corridors. Therefore, the indirect degree of a minority of O-D pairs on the HSR network would be much higher than those on the conventional rail network. Generally, we consider that high-speed trains only run on the HSR network in China [1].With the increasing circuity on the HSR network, people would choose conventional railways, highways, or air instead of high-speed railway. On the HSR network in mainland China completed at the end of 2014 (see **Fig 1**), the length of the shortest path between Chengdu and Xi’an is 2244 km, which passes through Chongqing North, Yichang East, Wuhan, and Zhengzhou Railway Stations; while the lengths of the paths between this particular O-D pair are about 714 and 866 km on the highway network and conventional rail network, respectively, and the spatial distance is only 510 km. It is unreasonable to operate high-speed trains between Chengdu and Xi’an because of the high path circuity.

For a given O-D, the shortest path is normally utilized as the high-speed train path. However, it is not reasonable to operate trains between any O-D pairs because some O-D pairs may have high indirect degree. Le Corbusier [2] concluded that a man selected the path with a lower indirect degree when he had a definite destination.

Indirect degree can be an evaluation criterion for train paths, but it has not been used in the research on train-path selection. Circuity, the ratio of path length to Euclidean distance, is used to measure the indirect degree, and has been widely applied in the research on urban roadways and public transit networks. On the basis of circuity, weighted circuity, which considers the amount of passengers, is used to measure how efficiently the transportation network is designed to meet the passenger demands (Lee [3], Giacomin and Levinson [4]). Levinson and El-Geneidy [5] randomly selected a number of O-D pairs in 20 metropolitan areas in the United States and calculated the circuity of the shortest paths between the O-D pairs. Meanwhile, they investigated some workers and calculated the circuity between their residences and work locations. They found the circuity of home-work pairs is lower than those of the random O-D pairs. After calculating the circuity of random O-D pairs in 51 well-known metropolitan areas in the United States, Giacomin and Levinson [4] indicated that circuity in an urban road network gradually increased over time, and they concluded that short trips are more circuitous than long trips by proposing a function describing the relevance between circuity and distance. Huang and Levinson [6] discovered that the circuity of the transit network was higher than that of the road network and indicated that circuity can explain mode choice share of commuters. Lee *et al*. [7] utilized circuity to analyze the transit network indirect degrees of five metropolises in Korea and found that metropolises possessed lower circuity than smaller cities. They also considered that population density significantly impacted the urban transit network circuity because of the strong relationship between passenger flow and circuity. Based on a wide range of GPS travel survey and road network data, Dhakar and Inivasan [8] developed a travel-path-selection model considering circuity and intersection. Poken [9] added the circuity to constraint conditions for the distribution path algorithm and found that circuity controls were more effective than standard least-cost insertion heuristics.

Circuity has also been used in the research of aviation networks. Russon and Hollingshead [10] demonstrated that reasonable hub planning could decrease the degree of passenger travel circuitousness and enhance the advantage of regional air transportation so as to avoid the loss of travelers to other modes of travel. Meilus [11] analyzed the impact of airline mergers and network adjustment on passenger travel efficiency by measuring the circuity of air routes. In the study of train-path-selection problems, Lv *et al*. [12] solved the problem of the inconformity between the pass ticket routes and real traveling routes by devising a new method based on train-route restriction to design a pass route algorithm.

In related research on transportation network analysis, efficiency, structural properties, robustness and redundancy of networks are areas of significant concern to researchers. Lee [3] introduced two comparative measures: Total Travel Time Degree of Circuity (or Competitiveness) and In-vehicle Travel Time Degree of Circuity (or Competitiveness) to examine the efficiency of transit network configurations. On this basis, simple average and weighted average are also used to measure how efficiently the transit network is designed to meet the passenger demands. Du *et al*. [13] studied the robustness of the Chinese Airline Network via the removal of nodes and connections and found that the Chinese Airline Network is not as redundant and robust as the Worldwide Airline Network in the high-degree targeted attack strategy. Zhang *et al*. [14] investigated the evolution of the Chinese airport network, including the topology, the traffic and the interplay between them and found that the main topological properties of the network were steady although there exists a dynamic switching process inside the network. Sen *et al*. [15] indicated that the Indian Railway network displays small-world properties based on complex network theory.

However, circuity has not yet been used to analyze HSR networks and high-speed train paths. Following the studies of air transport, we suppose that the directness of high-speed train paths has a great influence on attracting passengers along the route. More specifically, this paper will contribute to the study of HSR networks by answering the following questions: (1) How can we determine the reasonable threshold of circuity for train paths? (2) For a large HSR network, what methods can help the planning of train paths? (3) How can we identify passenger corridors in a large railway network? (4) What is the influence of HSR construction on railway transportation?

## Indicators and data

**Fig 2:**
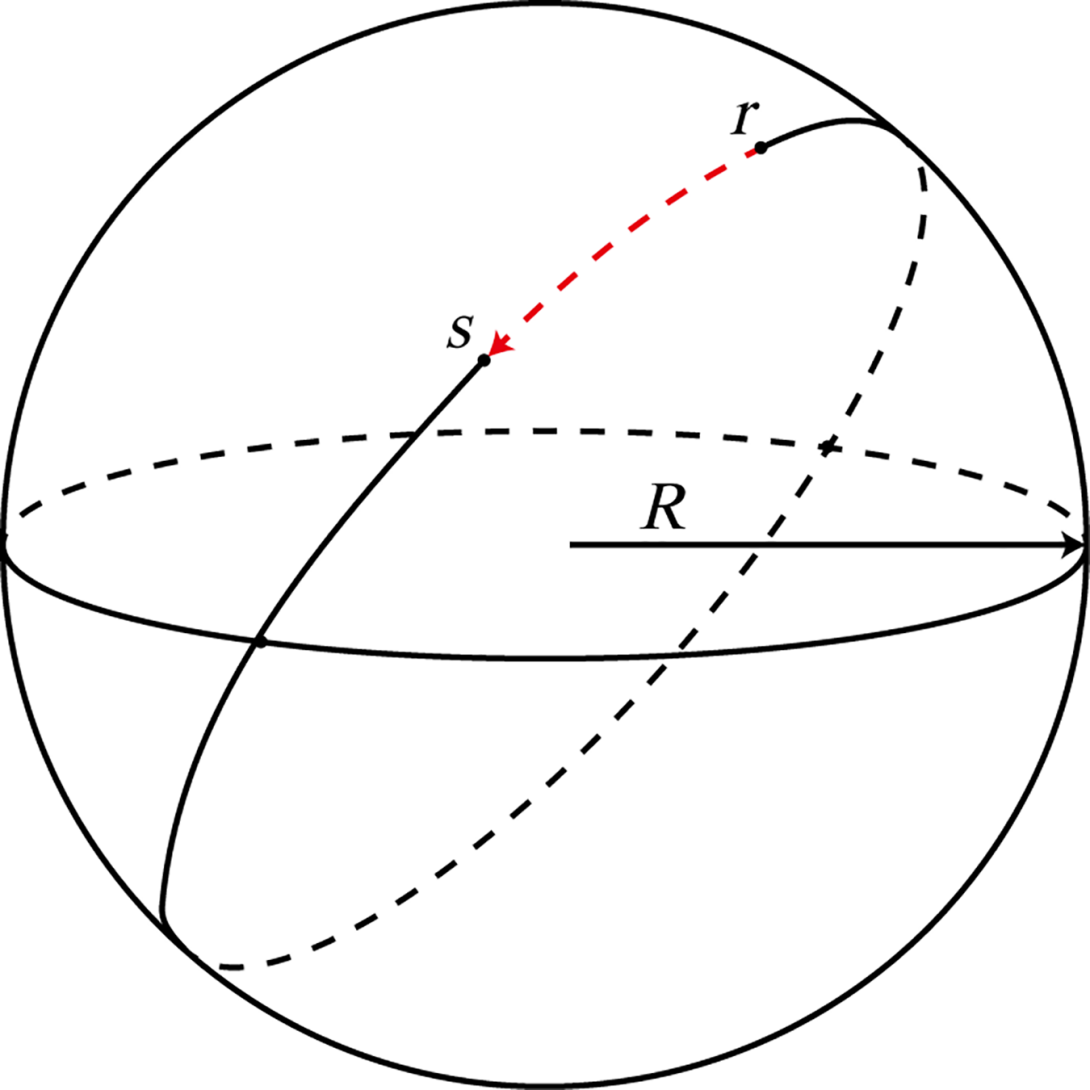
The great-circle distance between origin and destination.

Circuity in high-speed rail network can be obtained by the following formula:

$$C_{rs}=P_{rs}/D_{rs}$$ (1)

where *C*_*rs*_ is the circuity of the path between origin *r* and destination *s*, *P*_*rs*_ is the length of the shortest path between origin and destination, and *D*_*rs*_ is the great-circle distance. The great-circle distance is the shortest distance between origin and destination on the surface of a sphere. **Fig 2** shows the great-circle distance from origin *r* to destination *s*.

Since we need to analyze the circuity of a large number of O-D pairs, we adopt the Floyd-Warshall shortest path algorithm [16] to solve *P*_*rs*_ in formula (1). Circuity was generally defined as the ratio of the shortest network distance over the Euclidean distance between origin and destination (Levinson and El-Geneidy 2009 [5], Barthélemy 2011 [17], Giacomin and Levinson 2014 [4], Huang 2015 [6]). For HSR networks, the path distance is the length of the shortest path along the railway. Here the Euclidean distance is replaced by the great-circle distance because the length of a relatively long path would be underestimated by the method of Euclidean distance. Moreover, the distance between an O-D pair on the HSR network is always longer than it is on the urban transport network. Using Euclidean distance may impact the precision of results. On the other hand, we can easily obtain the longitudes and latitudes of origin and destination rather than their coordination in Euclidean space. The great-circle distance is calculated as follows [18]:

$$D_{rs}=R\;\cos^{-1}\left[\cos\;y_{r}\;\cos\;y_{s}\;\cos\left(x_{r}-x_{s}\right)+\sin\;y_{r}\;\sin\;y_{s}\right]$$ (2)

where *x*_*r*_,*x*_*s*_ ∈ [−*π*,*π*] are the longitudes of points *r*,*s* and $y_{r},y_{s}\in \left[0,\frac{\pi }{2}\right]$ are the latitudes of points *r*,*s*. There are $y_{r},y_{s}\in \left[0,\frac{\pi }{2}\right]$ rather than $y_{r},y_{s}\in \left[-\frac{\pi }{2},\frac{\pi }{2}\right]$ which means that points *r*,*s* have to be both within the same hemisphere. *R* is the Earth’s radius which is set as 6371.393 km (Ballou *et al*. 2002 [18]).

Meanwhile, we also utilize the concepts of circuity of railway lines and circuity of train paths. The circuity of a high-speed railway line is the ratio of rail-line length to the spherical distance between the origin and destination of the line, and the circuity of a train path is the ratio of path length to the spherical distance between the origin and destination of the train.

The study area is based on the HSR network in mainland China completed at the end of 2014. The network comprises 28 railway lines and 349 stations (operated by China Railways Corp.). Data on geographic coordinate of each station are provided by Baidu Coordinate Pick-up System. The 46 stations marked with solid circles in **Fig 1** are equipped with devices for the departure and arrival of trains (electric multiple unit depots, or EMUDs). All 46 EMUD stations are listed in **Table 1** [19]. We extracted the paths of operated trains from the high-speed train schedule which is sourced from China Railways Corp. in 2014.

**Table 1:** Stations equipped with EMUDs.

Beijing	Beijing South	Wuhan	Beijing West	Xi’an North
Hangzhou	Hangzhou East	Xiamen	Xiamen North	Guangzhou South
Fuzhou	Fuzhou South	Hankou	Harbin West	Shijiazhuang
Hefei	Hefei South	Jinan	Jinan West	Nanning
Nanjing	Nanjing South	Qindao	Qindao North	Taiyuan South
Shanghai	Shanghai Hongqiao	Shenyang	Shenyang North	Taiyuan
Tianjin	Tianjin West	Changchun	Changchun West	Chongqing North
Nanchang	Nanchang West	Dalian	Dalian North	Changsha South
Zhengzhou	Zhengzhou East	Chengdu	Chengdu East	Shenzhen North
Wuchang				

**Fig 3:**
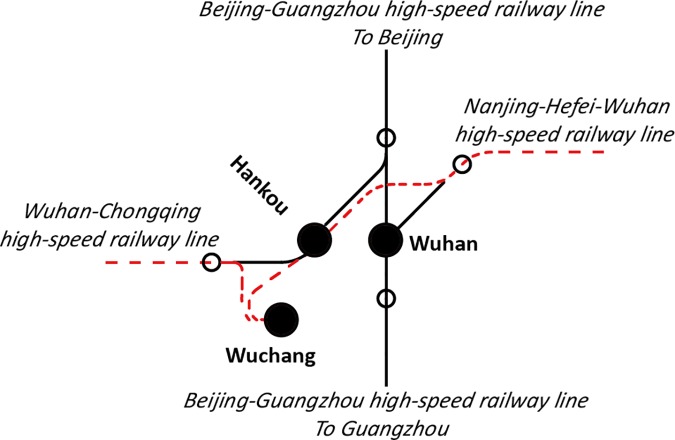
High-speed railway lines served by Wuchang Railway Station.

**Fig 4:**
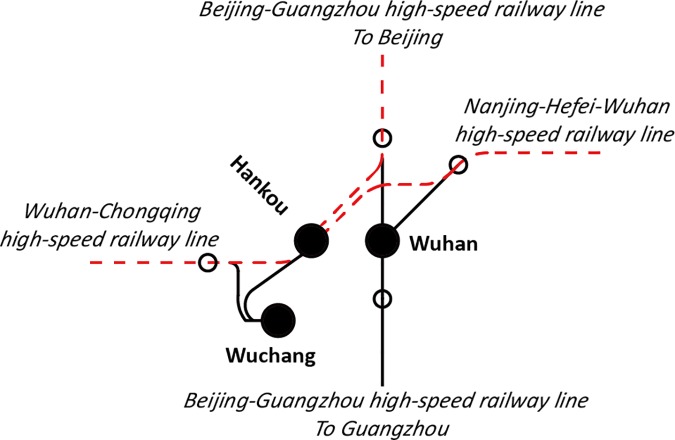
High-speed railway lines served by Hankou Railway station.

In addition, there may be more than one high-speed railway station in a city, and they have clear work assignments to access trains operated on different railway lines [20]. For example, there are three railway stations in Wuhan, i.e., Wuhan, Wuchang, and Hankou Railway Stations, which link the Beijing-Guangzhou, Nanjing-Hefei-Wuhan, and Wuhan-Chongqing high-speed railway lines, respectively. Wuhan Station can access trains operated on all three high-speed railway lines. Wuchang Station can access trains only operated on Nanjing-Hefei-Wuhan and Wuhan-Chongqing high-speed railway lines. Hankou Station cannot access trains from Guangzhou direction along Beijing-Guangzhou high-speed railway line. The work assignment of Wuchang and Hankou Railway Stations are shown by dotted lines in **Figs 3 and 4**, respectively.

With respect to reasonable train-path-selection problems, since each high-speed railway line is a reasonable passenger transit corridor, we mainly discuss the situation in which both the origin and destination are on different high-speed railway lines and equipped with devices for departure and arrival of trains. Owing to a large number of reasonable O-D pairs, many train paths lap over each other, and thus we cluster train paths according to their inclusion relationship.

In order to analyze the circuity of any O-D pairs in a HSR network, we mainly research the distribution of circuity and the distribution characteristics of some O-D pairs with high circuity. Furthermore, combining high-speed railway line planning and construction, we analyze the variation trend of circuity distribution in HSR networks.

## Circuity of high-speed train paths

High-speed trains can be classified as two types based on paths: one includes within-line trains (of which origin and destination are on the same line) and the other includes cross-line trains (of which origin and destination are on the different lines). We can separately solve the circuity of paths of these two types of high-speed trains. For the first type, we only have to calculate the circuity of each high-speed railway line. For the second, we need to calculate the circuity of train paths one by one.

The HSR network depicted in **Fig 1** consists of 28 railway lines (operated by China Railways Corp.); we can obtain the circuity of these lines (**Table 2**) using formula (1).

**Table 2:** Circuity of each high-speed railway line investigated in this paper (in descending order).

Name of railway line	Origin	Destination	Circuity
Jiaoji high-speed rail	Jinan	Qingdao	1.29
Longyan- Xiamen	Longyan	Xiamen	1.28
Datong-Xi’an (Partial)	Shijiazhuang	Weinan	1.26
Beijingnan-Shanghai	Beijingnan	Shanghai Hongqiao	1.24
Ningbo-Wenzhou-Fuzhou	Ningbo	Fuzhou	1.23
Nanchang-Fuzhou	Nanchang	Fuzhou	1.23
Chengdu-Chongqing	Chengdu	Chongqing	1.21
Beijing-Guangzhou	Beijing	Guangzhou	1.21
Nanning-Qinzhou	Nanning	Qinzhou	1.19
Nanjing-Hefei-Wuhan	Nanjing	Wuhan	1.16
Hefei-Bengbu	Hefei	Bengbu	1.16
Nanchang-Jiujiang	Nanchang	Jiujiang	1.16
Hangzhou-Xinhuang (Partial)	Hangzhou	Xinhuang	1.15
Dazhou-Chengdu	Dazhou	Chengdu	1.15
Guangzhou-Shenzhen	Guangzhou	Shenzhen	1.15
Zhengzhou-Baoji	Zhengzhou	Baoji	1.14
Hangzhou-Ningbo	Hangzhou	Ningbo	1.14
Hengyang-Liuzhou-Nanning	Hengyang	Nanning	1.14
Beijing-Shenyang	Beijing	Shenyang	1.13
Fuzhou-Xiamen	Fuzhou	Xiamen	1.13
Wuhan-Chongqing	Hankou	Chongqing	1.13
Shanghai-Nanjing	Shanghai	Nanjing	1.12
Nanjing-Hangzhou	Nanjing	Hangzhou	1.11
Changchun-Jilin	Changchun	Jilin	1.11
Xiamen-Shenzhen	Xiamen	Shenzhen	1.09
Shanghai-Hangzhou	Shanghai Hongqiao	Hangzhou	1.09
Harbin-Dalian	Harbin	Dalian	1.09
Nanning-Guangzhou (Partial)	Nanning	Wuzhou	1.08

**Fig 5:**
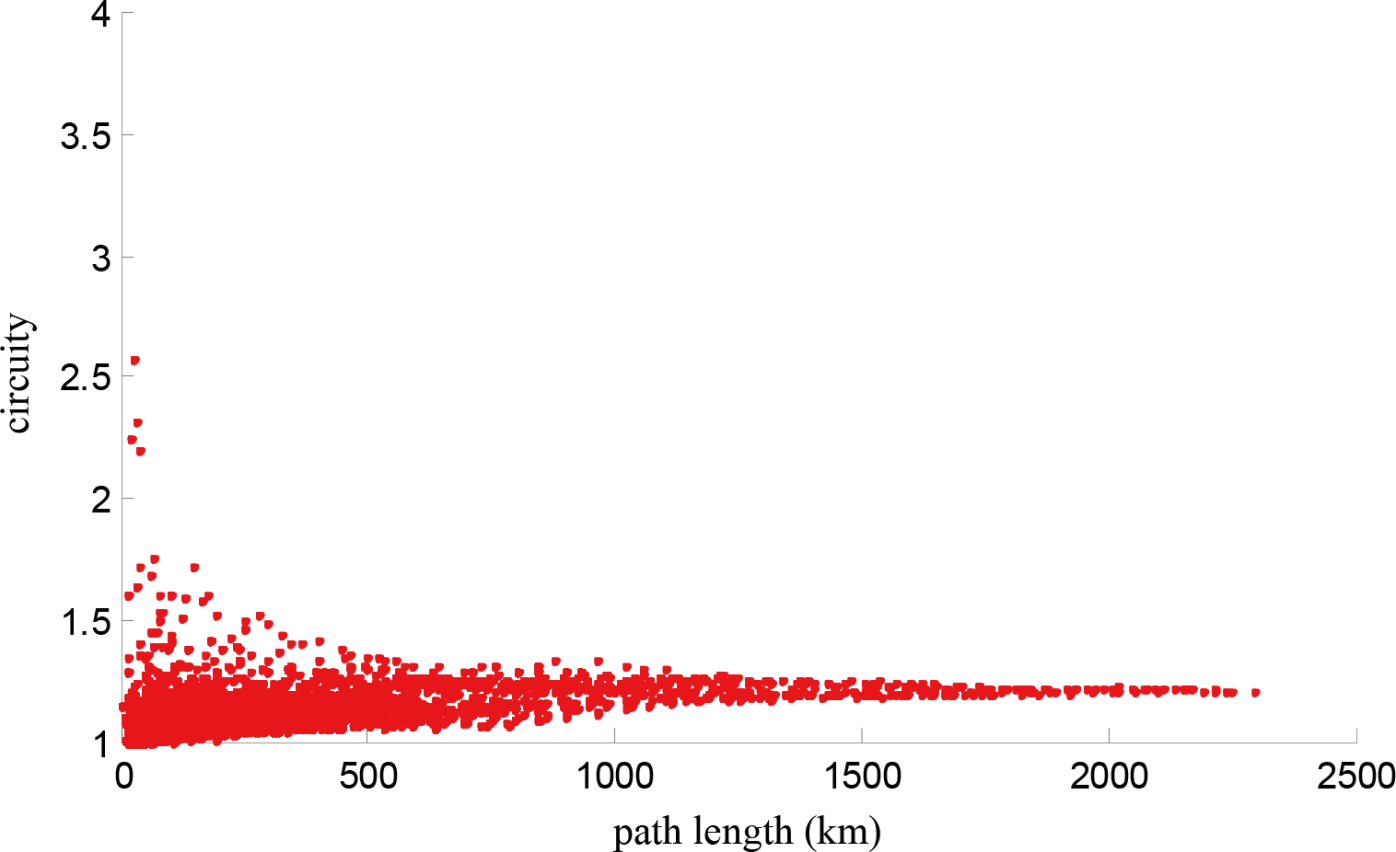
Relationship between path length and circuity of any two points on each high-speed railway line.

According to **Table 2**, the maximum circuity of the railway lines investigated is 1.29, the minimum is 1.08, and the average is 1.16. In general, high-speed railway lines are not circuitous. The circuity of Jiaoji HSR is the highest because of the terrain of Shandong peninsula. Similarly, the circuity of any part of the railway lines is also low. The relationship between the path length and circuity of each O-D pair on each high-speed railway line is illustrated in **Fig 5**. From **Fig 5**, we observe that the circuity converges in the interval [1,1.5].

In 2014, except for interurban railway lines, the high-speed trains in operation in China run on 292 train paths [21]. The circuity of train trip G7376/G7377 from Jiangshan Railway Station to Hefei South Railway Station, which passes through Hangzhou East, Shanghai Hongqiao, and Nanjing South Railway Stations, is the highest at 2.48. On the other hand, the lowest is 1.03, corresponding to train trip G7282 from Hefei Railway Station to Huainan East Railway Station, which passes through Shuijiahu Railway Station. The average circuity of the entire paths on this HSR network is 1.37.

**Fig 6:**
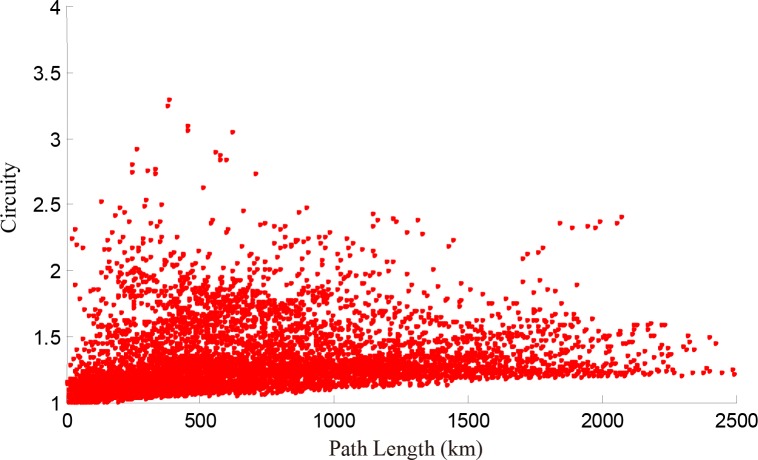
Relationship between path length and circuity of path between any two points on the operated train path.

For all high-speed train paths, the relationship between path length and circuity of each O-D pair is shown in **Fig 6**. The figure indicates that the maximum circuity of subpaths is obviously higher than that of train paths (2.48), the main reason being that there is a break angle as a part of a train path with a low circuity.

## Searching and clustering of train paths

### Determine the circuity threshold of train-paths

According to the analysis of circuity of high-speed railway lines and the paths of trains in Section 3, the average circuity of train paths is higher than that of railway lines. We set the maximum circuity of train paths, i.e., 2.48, as the threshold value, and under this constraint we search the reasonable train paths to form the train-path sets.

In contrast, the circuity of many urban road networks and public transit networks is lower than 2.48. For example, Newell [22] indicated that the circuity of the path between a random O-D pair is approximately 1.2 in a city. The national standard *Urban Road Traffic Planning and Design Specification* issued by the China State Bureau of Technical Supervision and the Ministry of Construction in 1995 sets a limit that the circuity of a bus path cannot exceed 1.4 [23].

### Clustering of train paths

Since each subpath of a train path is reasonable, a new path can also be formed by an existing path and an extra branch path. Under the constraint of the given threshold value of path circuity, the number of train paths is too large, and their description would be complicated. In order to simplify the expression of cross-line train path sets, we should cluster all of the reasonable train paths.

We cluster the paths mainly on the basis of overlap ratio of train paths. In this paper, the train path between two nodes is the shortest path and these shortest paths are often exclusive, so the overlap ratio of two train paths can be described by the relationship between their node sets.

We denote *N*_*rs*_ as the node set of a path *P*_*rs*_ between the O-D pair (*r*,*s*), and its node number is |*N*_*rs*_|. For path node sets *N*_*rs*_ and *N*_*ij*_, accurate clustering can be expressed by the inclusion relation between the two path node sets.

If *N*_*ij*_ ⊆ *N*_*rs*_, then the train path *P*_*ij*_ belongs to the class represented by train path *P*_*rs*_.

Except for the accurate clustering, some error is allowed. Error can be described in terms of two conditions, i.e., absolute error and relative error. Absolute error means the number of nodes in *N*_*ij*_ that do not belong to *N*_*rs*_ and the error cannot exceed the threshold value Δn:

$$|N_{ij}\backslash N_{rs}|\leq \Delta n$$ (3)

Relative error means the ratio of number of nodes in *N*_*ij*_ that do not belong to *N*_*rs*_ and the ratio does not exceed the threshold value *ε*:

$$\left|N_{ij}\backslash N_{rs}\right|/\left|N_{ij}\right|\leq \varepsilon$$ (4)

When sets *N*_*rs*_ and *N*_*ij*_ satisfy either formula (3) or (4), path *P*_*ij*_ can be merged into the class represented by train path *P*_*rs*_.

For instance, the node set of path *P*_1,12_ is *N*_1,12_ = {1,2,3,4,5,6,7,8,9,10,11,12}, the node set of path *P*_2,15_ is *N*_2,15_ = {2,3,4,5,6,7,13,14,15}, Δ*n* = 3 and *ε* = 0.25. Although node sets *N*_1,12_ and *N*_2,15_ can be regarded as node sequence sets, we do not involve the node order when calculating absolute and relative errors. Obviously, |*N*_2,15_| = 9; therefore, absolute error |*N*_2,15_\*N*_1,12_| is 3 and relative error |*N*_2,15_\*N*_1,12_|/|*N*_2,15_| is 3/9 ≈ 0.33. Since the absolute error satisfies formula (3), *P*_2,15_ can be merged into the class represented by *P*_1,12_.

We designate the representative in one path class as a center path, which has the most nodes in this class. Clustering paths can be carried out in descending order of the number of path nodes; the detailed clustering procedure is as follows:

Step 1: Add all paths to the path set Ω to be clustered, and arrange the paths in the descending order of the number of path nodes.

Step 2: Choose the path with the largest number of nodes from Ω to be the center path of one path class, determine whether other paths belong to this class by formula (3) or (4), and delete the paths in the new path class from Ω.

Step 3: Repeat Step 2 until Ω is empty.

## Searching and clustering train paths in Chinese HSR network

**Fig 7:**
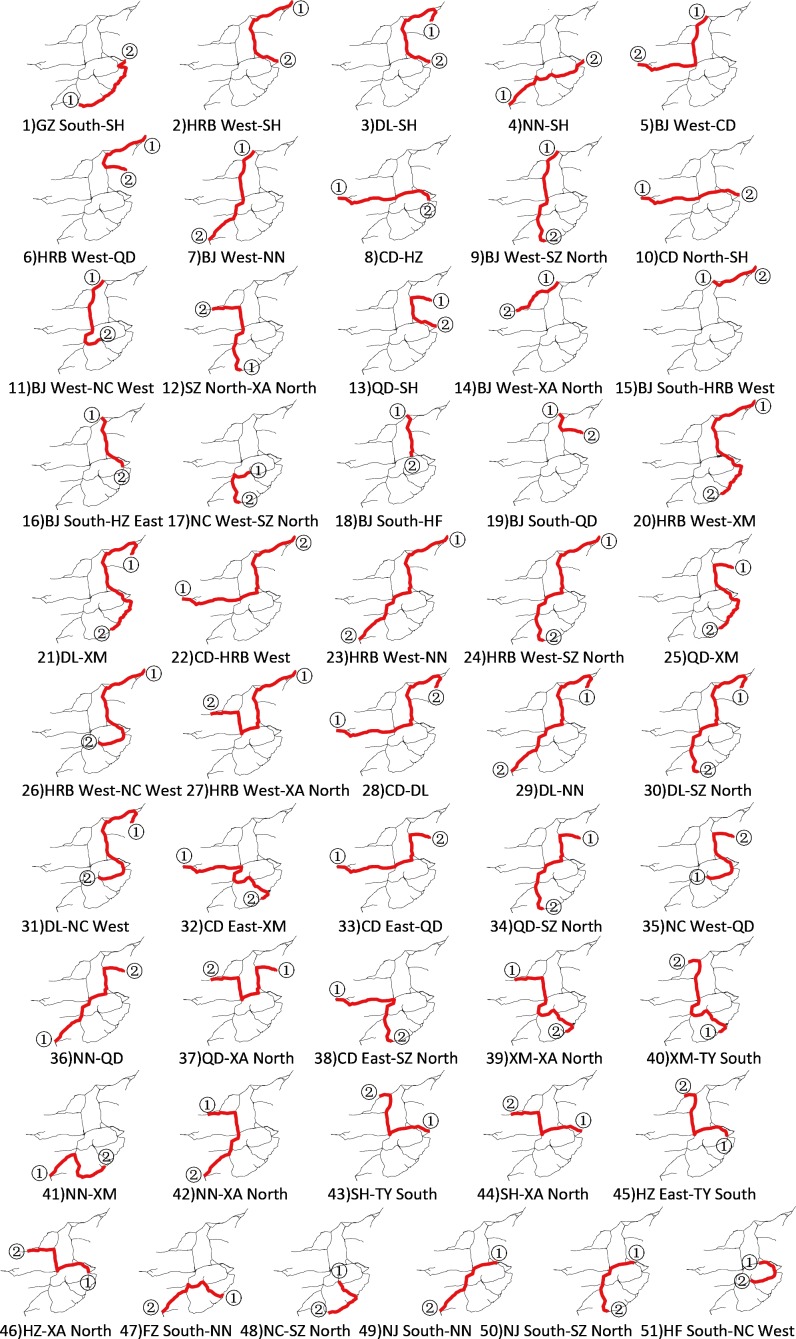
Illustration of the 51 center passenger transit corridors.

In this section, we take the HSR network in China (except for the Hainan East Ring Line and some intercity railway lines) depicted in **Fig 1** as an example. Combining the stations in the same city, we obtain 26 nodes with the ability to access the departure and arrival of trains, and those form 325 O-D pairs, of which 274 are cross-line pairs. We calculate the circuity of these O-D pairs using formula (1), and find that the circuity of 196 cross-line O-D pairs does not exceed 2.48. We set the maximum absolute error Δ*n* to 3 and the maximum relative error *ε* to 0.05. Using the clustering method described in Sec. 4, we obtain the 51 classes of paths illustrated in **Fig 7**, which consists of 51 subfigures, where ① & ② denote the origin and destination of each center path. The black solid lines denote the HSR network and the bold red lines the center paths (passenger transit corridors). In **Fig 7**, there are already through trains running on the passenger transit corridors labeled 1 to 19, while few through trains are operated on the passenger transit corridors labeled 20 to 51. As long as there is adequate passenger demand, we can operate trains on these alternative passenger transport corridors (labeled 20 to 51).

The longest center path measures 3705 km, which is constrained by the standard that electric multiple units must stop to be inspected and repaired at Level One after running 4000 km, and the two terminal stations of this path must have EMUDs. The shortest center path measures 819 km, exceeding the full mileage of some trains. If we change the circuity threshold value to 2.06 (the average of the circuity of the cross-line trains operated), many paths to some major cities, such as Qingdao and Dalian, would be excluded.

However, in the practical operation, some trains do not run along the shortest path but pass through some significant cities. These paths are as follows:

1. The Ning Hang high-speed railway line connects Hangzhou East and Nanjing South Railway Stations, and the shortest path between these two cities on the HSR network is 256 km, but the length of some train paths between these two cities reaches 470 km. For example, some paths that start from Hangzhou East Railway Station, pass through Shanghai Railway Station, and then arrive at Nanjing South Railway Station. These particular cases result from the significant position of Shanghai in the entire network.
2. The shortest path from Beijing West Railway Station to Xi’an North Railway Station is the Da Xi high-speed railway line via Shijiazhuang and Taiyuan Railway Stations, which is 1092 km. However, the path of the majority of trains from Beijing West Railway Station to Xi ‘an North Railway Station passes through Jing Guang and Zheng Xi high-speed railway lines (via Zhengzhou East Railway Station), and the total length is 1216 km. The main reason behind this is that the designed speed of Jing Guang and Zheng Xi high-speed railway lines (350 km/h) is higher than that of Da Xi high-speed railway line (250 km/h), although other administrative reasons cannot be excluded.
3. Between Hangzhou East Railway Station to Guangzhou South Railway Station, some of trains run along Hu Kun and Jing Guang high-speed railway lines via Changsha South Railway Station, but other portion of trains named “High-speed railway EMU with sleepers” run along Hang Fu Shen high-speed railway lines. The train path of the former is the 65 km shorter than the latter. Two paths are designed for 350 km/h and 250 km/h respectively.

**Fig 8:**
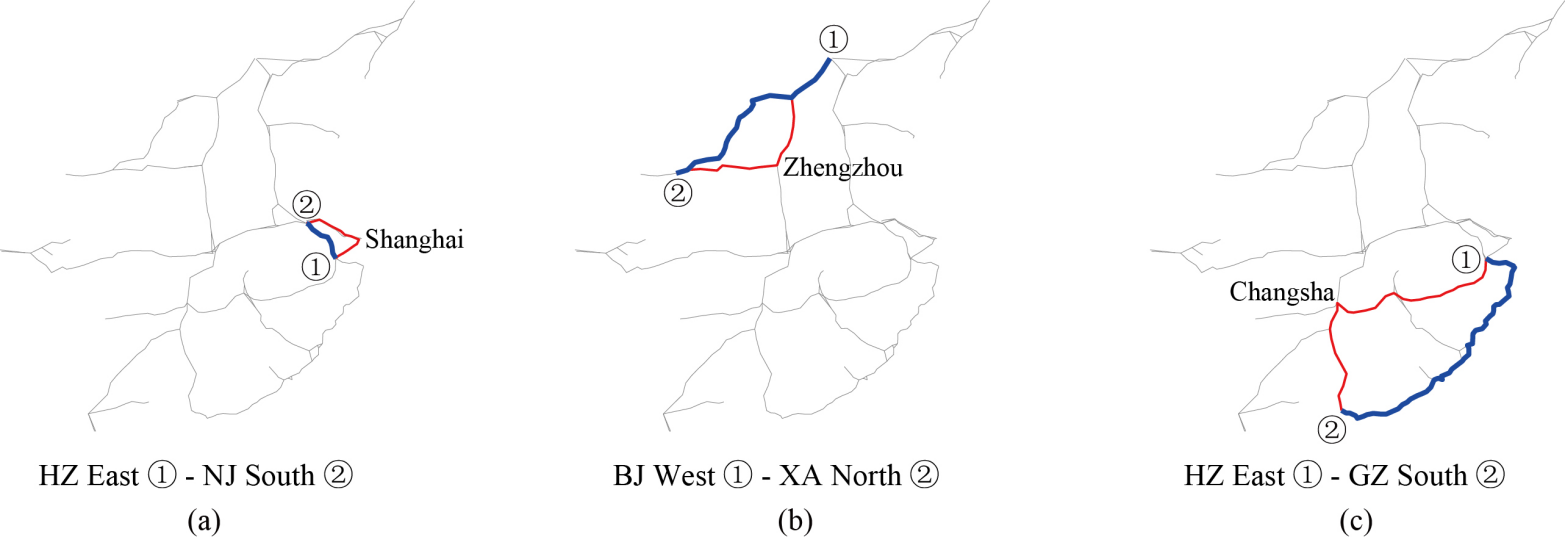
Illustration of the shortest and nonshortest paths of three point pairs in China’s high-speed rail network.

The specific paths in the three above situations are shown in **Table 3**, and **Fig 8** is the table’s complementary diagram, in which bold lines show the shortest paths and fine lines represent practical paths.

**Table 3:** Cases in China for which the paths of trains are not the shortest during practical operation.

O-D	Shortest path	Real train path
**HZ East−NJ South**	HZ East−YX−NJ South	HZ East−SHHQ−NJ South and HZ East−YX−NJ South
**BJ West−XA North**	BJ West−SJZ−TY South−XA North	BJ West−ZZ East−XA North
**HZ East–GZ South**	HZ East–FZ South–GZ South	HZ East–CS North–GZ South and HZ East–FZ South–GZ South

In conclusion, during the process of HSR train-path searching, the reasonable shortest paths between O-D pairs should be mainly selected. However, in several cases, the shortest path may not be the practical option so that the train can pass through an important metropolis or the superior railway lines.

## Circuity in Chinese HSR network

HSR networks are the foundation of train-path selection. Hence, from the point of analyzing circuity of any O-D pairs in the network, we evaluate the efficiency of a HSR network structure.

**Fig 9:**
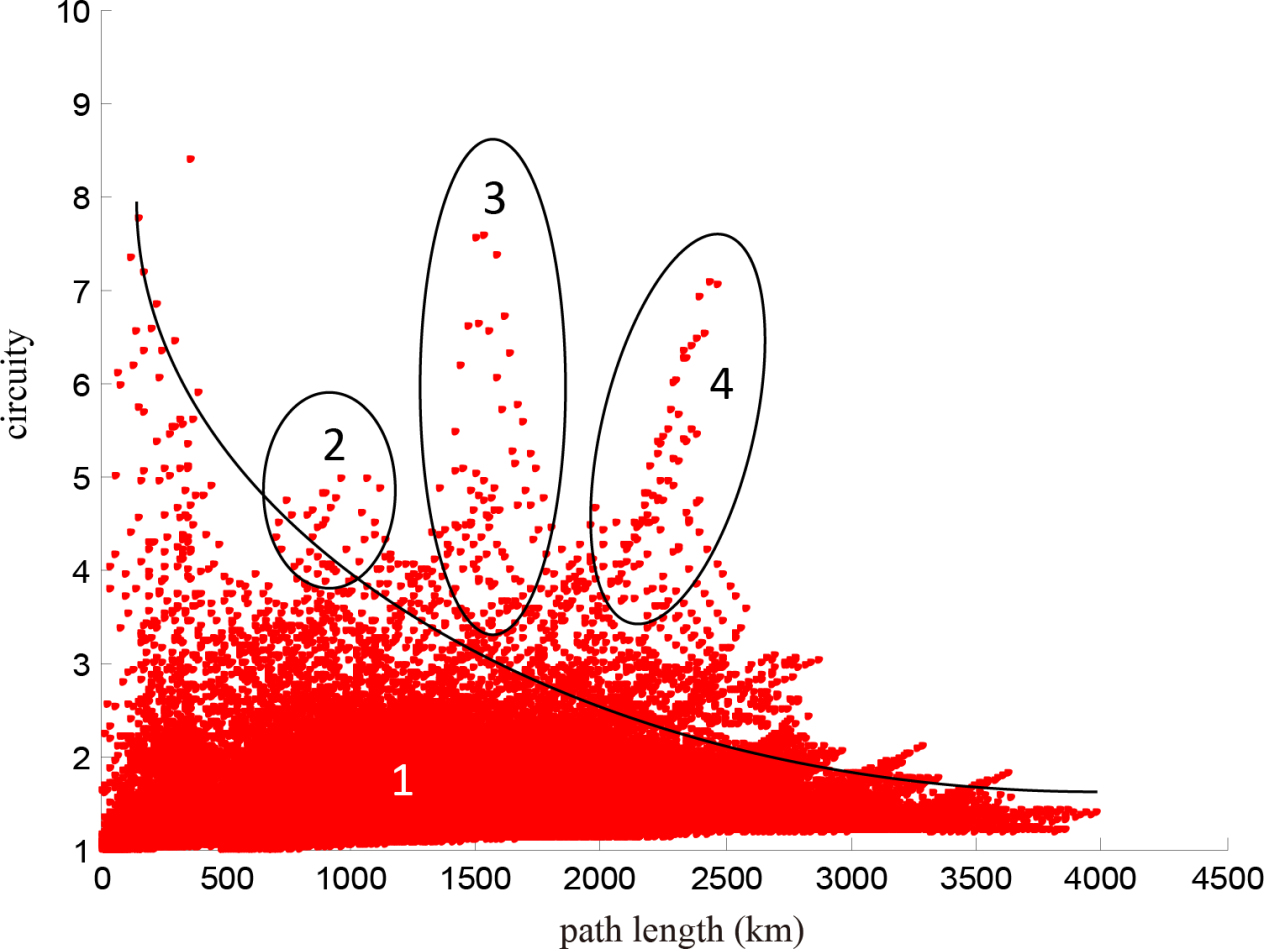
Relationship between length of the shortest path and circuity of any two points on the network.

**Fig 10:**
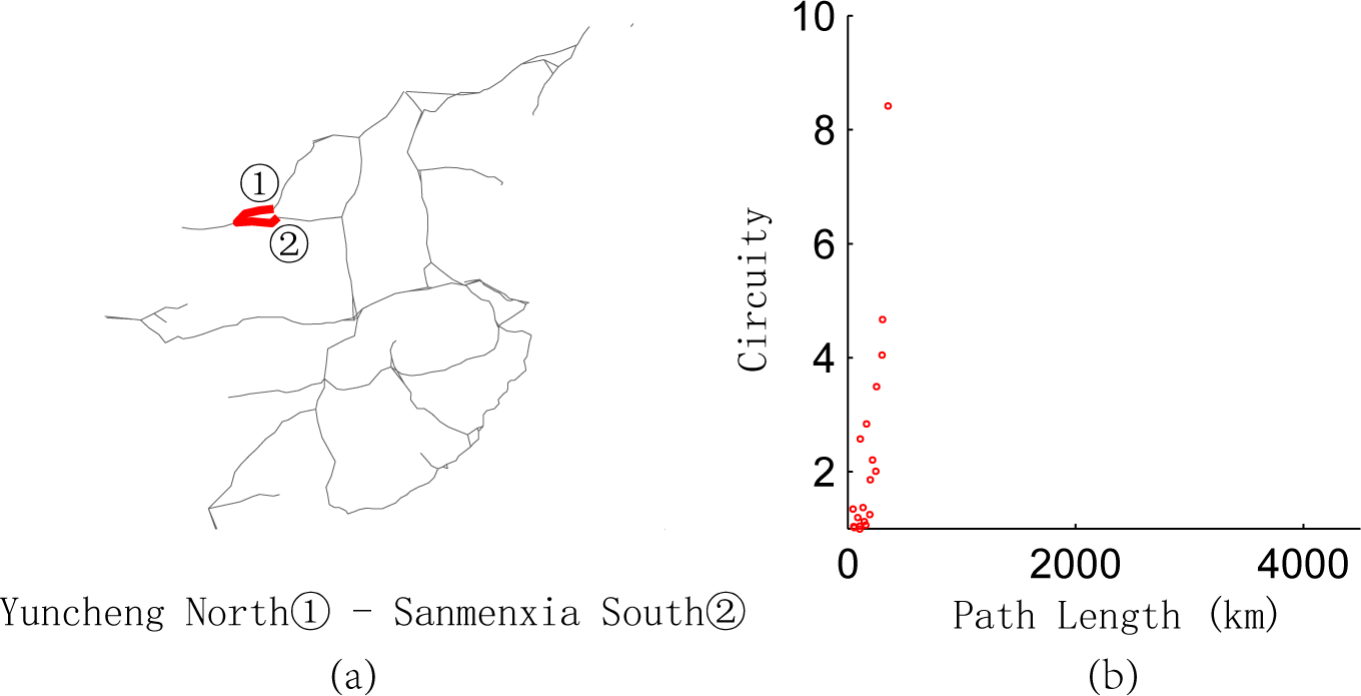
Path from Yuncheng North Railway Station to Sanmenxia South Railway Station and circuity analysis.

**Fig 11:**
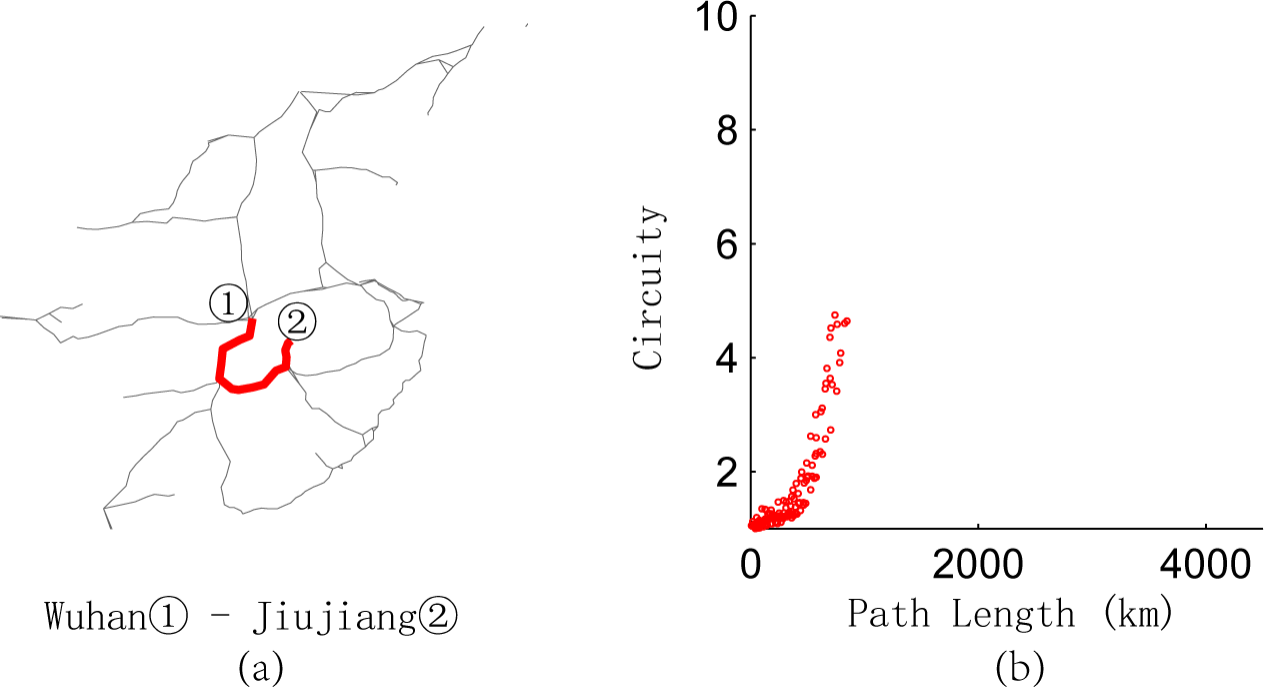
Path from Wuhan Railway Station to Jiujiang Railway Station and circuity analysis.

**Fig 12:**
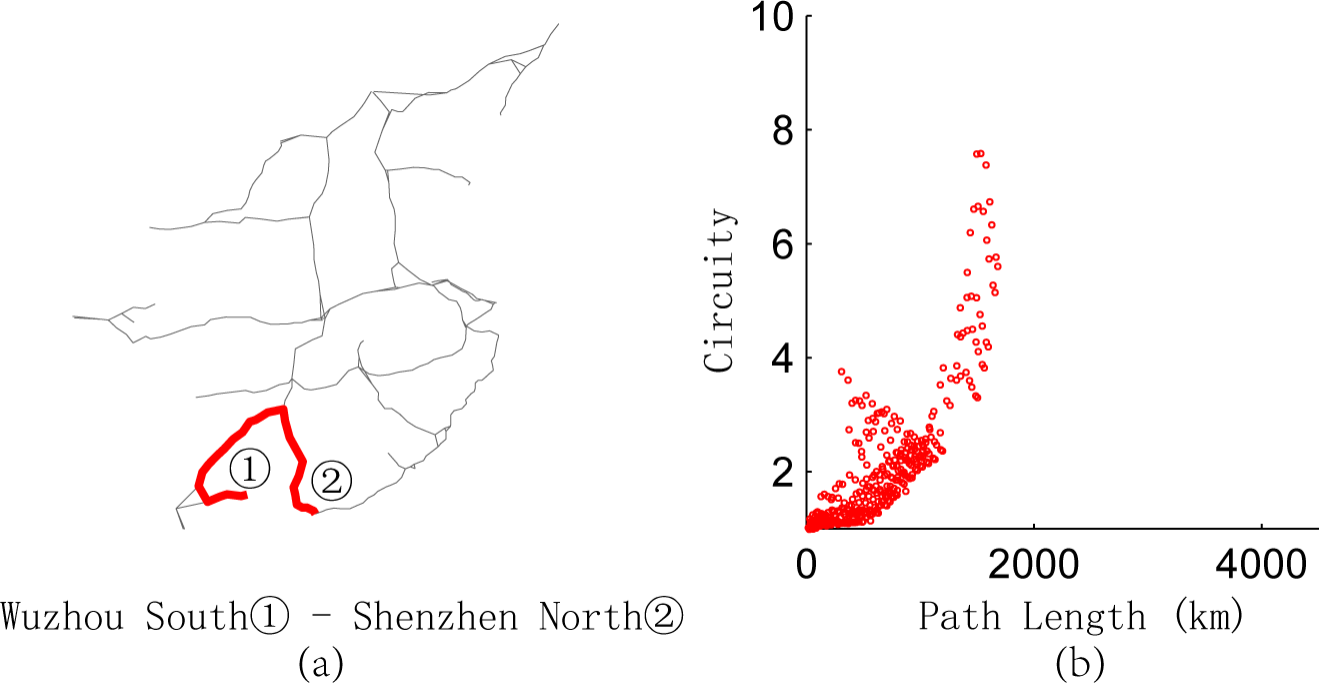
Path from Wuzhou South Railway Station to Shenzhen North Railway Station and circuity analysis.

**Fig 13:**
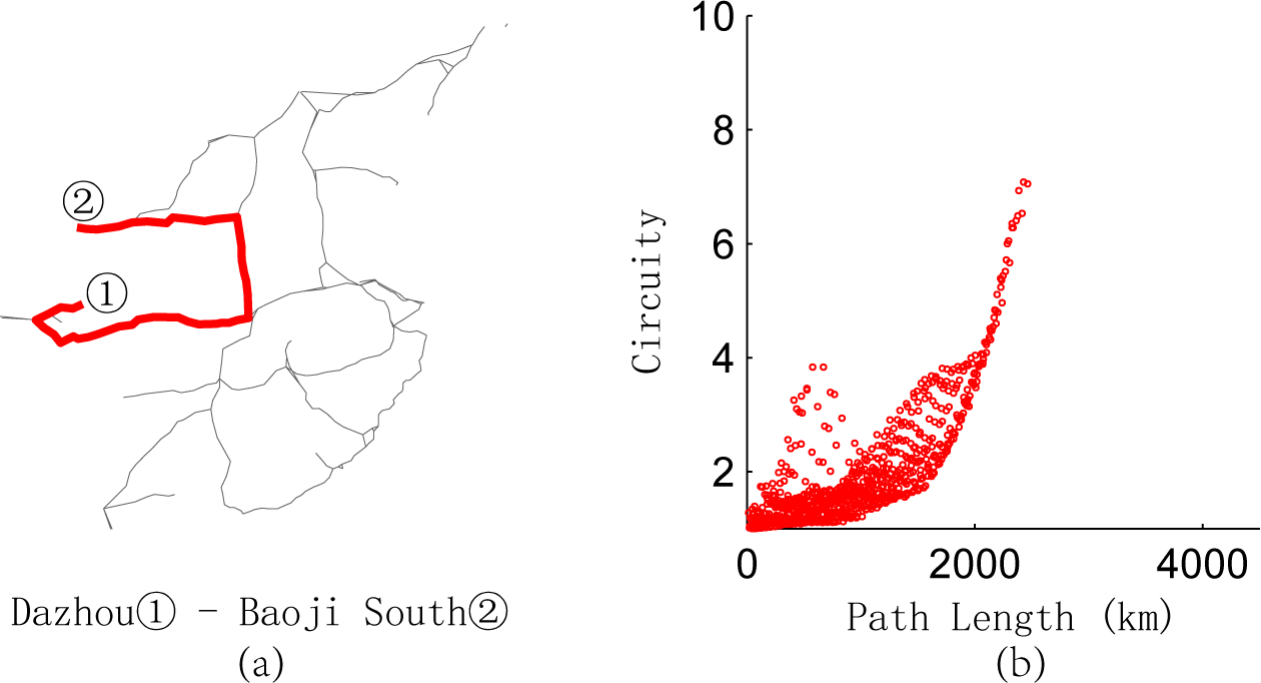
Path from Dazhou Railway Station to Baoji South Railway Station and circuity analysis.

We calculate the length of the shortest path and circuity of any O-D pairs in the network represented in **Fig 1** and depict the relationship between them in **Fig 9**, which is divided into four parts. Most points converge in part 1, which shows integrally that the longer the paths are, the lower the circuity. This result is consistent with the conclusion of Giacomin and Levinson [4] for urban road networks. Owing to the existence of break angle segments, there are some O-D pairs with short paths but high circuity in part 1. For example, the path from Yuncheng North Railway Station to Sanmenxia South Railway Station [**Fig 10(A)**] goes through the intersection of two railway lines, so the path between any two nodes on this path has short length but high circuity [**Fig 10(B)**]. Parts 2–4 of **Fig 9** are relatively special because of the long path length and high circuity. We find that the node pairs in these three parts are mainly distributed on paths from Wuhan Railway Station to Jiujiang Railway Station, from Wuzhou South Railway Station to Shenzhen North Railway Station, and from Dazhou Railway Station to Baoji South Railway Station [**Figs 11(A)–13(A)**]. Moreover, the relationships between the circuity and length of the path between any two points on these three paths are shown in **Figs 11(B)–13(B)**, respectively. The long distances and high indirect degrees of these paths result from the lack of direct railway lines.

**Fig 14:**
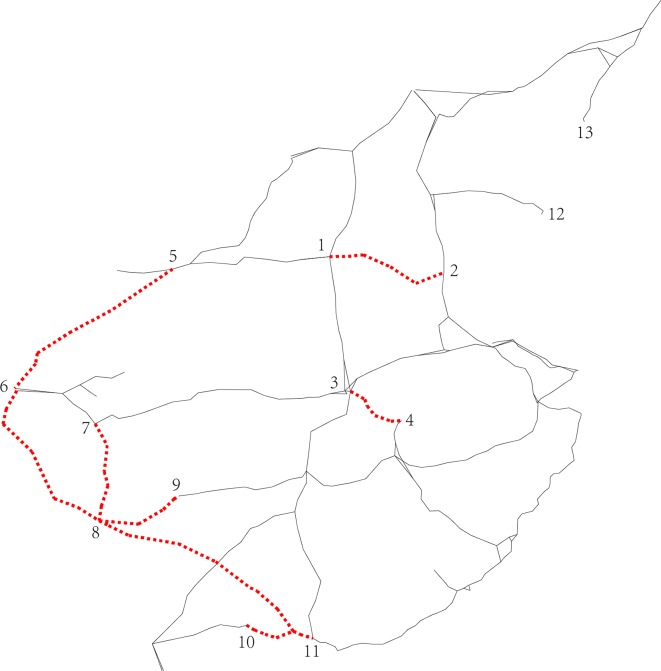
Planned network of high-speed rail in China.

**Fig 15:**
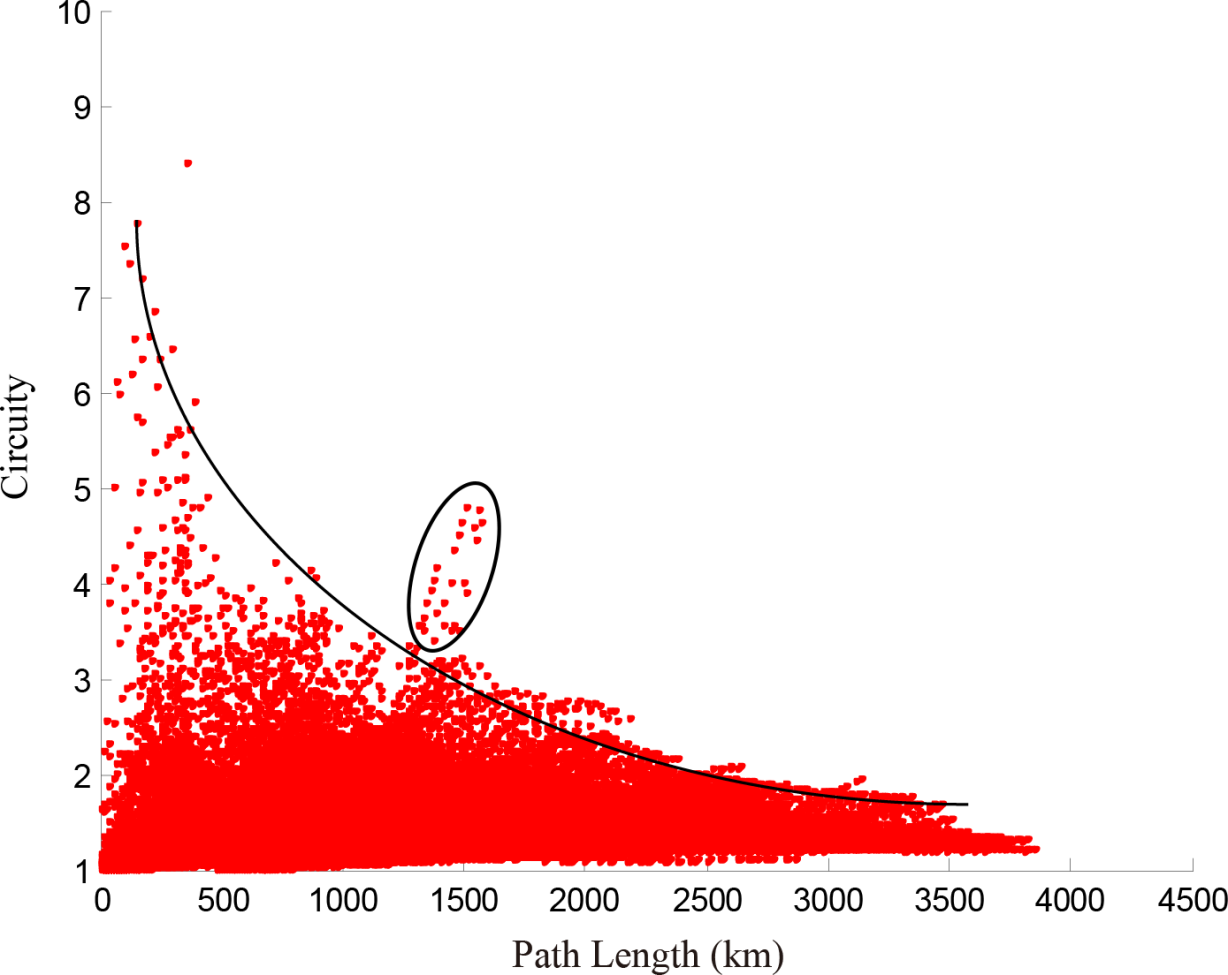
Relationship between path length and circuity of any two points in the planned network (see Fig 14).

In terms of the short-range design of the HSR network in China (adjusted in 2008, National Development and Reform Commission) [24], Zheng Xu (1 to 2), Wu Jiu (3 to 4), Xi Cheng (5 to 6), Cheng Gui (6 to 8), and Nan Guang high-speed railways (10 to 11) are in planning or under construction, and are shown in **Fig 14**, where new lines are shown by dashed lines. **Fig 15** illustrates the relationship between path length and circuity of the path between any two points on the network presented in **Fig 1**. In contrast to **Fig 9**, the circuity in **Fig 15** decreases dramatically due to these planning lines, and parts 2–4 disappear. It is notable that there is still a convex part (in the black circle) that represents the O-D pairs on the path from Qingdao Railway Station to Dalian Railway Station (12 to 13). These two cities, located in two peninsulas of China, i.e., the Shandong and Liaodong peninsulas, are close in space but separated by the sea. There is no sea-crossing bridge or subsea tunnel between these two cities, so it is difficult to operate high-speed trains between them. With the increase of the density of a HSR network, the circuity between any two nodes increases at first and then decreases with path length. From the above discussion, we conclude that the structure of China's HSR network tends to be completed.

## Conclusions

This paper adopted circuity, the ratio of network to Euclidean distance, to analyze HSR network and high-speed train paths. After comparing train paths circuity with that of road network and bus routes, we set the maximum path circuity of operated high-speed trains in China as the threshold value. Under constraint of circuity threshold value, we can search all of the reasonable train paths on the network. Then we propose a clustering method based on the inclusion relation which is defined as a range of absolute or relative error between each two paths. By using a clustering method according to the inclusion relation between each two paths, we simplify the expression of a large number of reasonable train paths. More importantly, we also obtain a series of passenger transit corridors which are center paths of train path classes. High-speed trains can be operated along these corridors when there are adequate passenger demands.

Circuity of high-speed railway lines is quite low (from 1.08 to 1.29) in the Chinese HSR network, which means that each HSR line can provide a reasonable train path. For both the constructed HSR network and the planned HSR network in China, the circuity of any O-D pairs are examined. The results show that the longer the paths, the lower the circuity, which is consistent with the results for urban road networks and transit networks found in preceding research. The circuity of the planned HSR network with more HSR lines is lower than the constructed HSR network, which demonstrates that the structure of China's HSR network tends to be complete. Meanwhile, we also analyze the reason that a few O-D pairs have a relatively high circuity value. These O-D pairs show that if we build HSR lines between these zones, the construction of the network can be improved significantly.

## Supporting information

S1 Data Basic data about the Chinese HSR network. (XLSX)

S2 Data Longitude and latitude of the nodes in the network. (XLSX)

S1 Table The result of circuity analysis of train paths. (XLSX)

S2 Table 51 classes of train paths. (XLSX)

S3 Table The result of circuity analysis of the operated network. (XLSX)

S4 Table The result of circuity analysis of the Chinese planned network. (XLSX)
